# Investigating the Ultrafast Dynamics and Long-Term Photostability of an Isomer Pair, Usujirene and Palythene, from the Mycosporine-like Amino Acid Family

**DOI:** 10.3390/molecules27072272

**Published:** 2022-03-31

**Authors:** Abigail L. Whittock, Jack M. Woolley, Nazia Auckloo, Christophe Corre, Vasilios G. Stavros

**Affiliations:** 1 Department of Chemistry, University of Warwick, Coventry CV4 7AL, UK; abbie.whittock@warwick.ac.uk (A.L.W.); jack.woolley@warwick.ac.uk (J.M.W.); nazia.auckloo@warwick.ac.uk (N.A.); c.corre@warwick.ac.uk (C.C.); 2 Analytical Science Centre for Doctoral Training, Senate House, University of Warwick, Coventry CV4 7AL, UK; 3 Warwick Integrative Synthetic Biology Centre, School of Life Sciences, University of Warwick, Coventry CV4 7AL, UK

**Keywords:** mycosporine-like amino acids, usujirene, palythene, ultrafast spectroscopy, photophysics, photochemistry

## Abstract

Mycosporine-like amino acids are a prevalent form of photoprotection in micro- and macro-organisms. Using a combination of natural product extraction/purification and femtosecond transient absorption spectroscopy, we studied the relaxation pathway for a common mycosporine-like amino acid pair, usujirene and its geometric isomer palythene, in the first few nanoseconds following photoexcitation. Our studies show that the electronic excited state lifetimes of these molecules persist for only a few hundred femtoseconds before the excited state population is funneled through an energetically accessible conical intersection with subsequent vibrational energy transfer to the solvent. We found that a minor portion of the isomer pair did not recover to their original state within 3 ns after photoexcitation. We investigated the long-term photostability using continuous irradiation at a single wavelength and with a solar simulator to mimic a more real-life environment; high levels of photostability were observed in both experiments. Finally, we employed computational methods to elucidate the photochemical and photophysical properties of usujirene and palythene as well as to reconcile the photoprotective mechanism.

## 1. Introduction

Mycosporines and mycosporine-like amino acids (both termed MAAs herein) are molecular chromophores with a strong ultraviolet (UV) absorption across both UVA (400–315 nm) and UVB (315–280 nm) spectral regions [1,2,3]. MAAs are small organic molecules that are often produced in cyanobacteria, fungi and algae, which then, in turn, leads to their accumulation in numerous marine mammals [1,4,5]. Due to this strong UV absorption, and their accumulation in their biological hosts when exposed to UV, they are believed to provide photoprotection alongside several other proposed functions, including as antioxidants [1,4,6,7,8,9]. The biosynthesis of MAAs was long believed to take place via a shikimate pathway evidenced by several inhibitor and labelling studies [10,11,12]; however, more recent work by Balskus and Walsh identified a gene cluster in cyanobacteria and concluded that MAA biosynthesis occurs via a pentose phosphate pathway [13]. Following this work, a study found that both pathways are in fact used in MAA biosynthesis and that the shikimate pathway is predominant when MAA synthesis is induced by UVA radiation [14].

In a broad sense, MAAs have two general structures, either that of a cyclohexenone core, defined as mycosporines, or that of a cyclohexenimine core termed mycosporine-like amino acids [6]. We recently reported, to the best of our knowledge, the first ultrafast spectroscopy on two common MAAs, shinorine and porphyra-334, which provided new insight into how MAAs safely remove excess absorbed energy from UV radiation [15]. This work, along with the complementary experimental literature, revealed that MAAs, such as palythine, porphyra-344 and shinorine, dissipate the majority of gained electronic energy from the absorbed UV photon as vibrational motion to the surrounding solvent environment, i.e., as heat [15,16,17]. These findings correlate well with theoretical calculations showing that the electronic excited state potential energy surfaces for palythine and porphyra-344 promote relaxation back to the electronic ground state through an easily accessible conical intersection (CI) [18,19,20]. The motion from the Franck-Condon geometry to the CI has been identified computationally as a planar to non-planar ring flexing movement [18,20,21,22].

**Figure 1:**
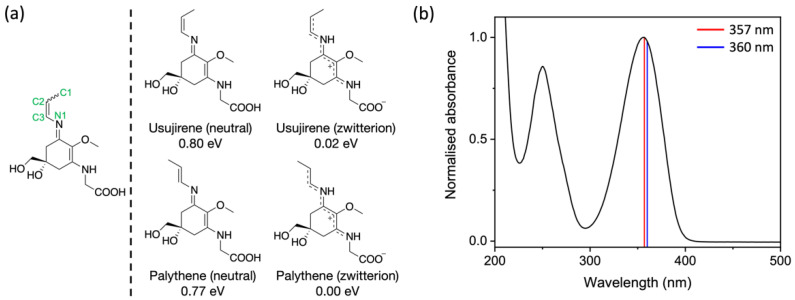
(**a**) Left: Structure of usujirene and palythene with relevant atom numbers labelled. Right: Chemical structures of usujirene and palythene both displayed as their neutral and zwitterionic conformers. Their relative energies calculated at the density functional theory (DFT)/PBE0/6-311++G** level in implicitly modelled water are reported with respect to zwitterionic palythene, the lowest energy conformer. (**b**) UV-visible spectrum of the usujirene/palythene aqueous solution with their respective peak absorptions given as vertical lines; red for usujirene and blue for palythene [4].

One particular pair of MAAs with a strong UVA absorbance (ε ≈ 50,000 dm^3^ mol^−1^ cm^−1^) is that of usujirene and its geometric isomer palythene [23], see Figure 1a for their structures. Usujirene and palythene are distinct in the MAA family due to the presence of a double bond conjugated to the core cyclohexenimine structure, the photoisomerisation of which has been previously studied by Conde et al. through steady-state spectroscopy and HPLC [24]. Their work showed that the quantum yield of photoisomerisation of usujirene to palythene is 1.71 ± 0.13 × 10^−5^ following irradiation with a monochromatic beam centered at 366 nm [24]. In addition to this, the authors also show the photodecomposition quantum yield of usujirene to be 2.86 ± 0.8 × 10^−5^, again highlighting the impressive photostability of this MAA [24].

Given the current demand for natural or nature-inspired cosmetics and that the present literature implies MAAs are ideal candidates as UV filters and antioxidants [7,15,16,17,18,19,20,21,24,25], there are several products on the market that contain some MAAs which are mostly targeted as sun protection factor (SPF) boosting and anti-photoaging formulations [26,27,28,29]. However, there is still a lot to learn regarding their photoprotection properties and biosynthetic assembly; such knowledge will undeniably benefit the future development of biomimetic UV filters inspired by MAAs.

Here, we extracted and purified usujirene and palythene from the red algae *Palmaria palmata* (*P. palmata*). We studied the isomer pair using femtosecond transient absorption spectroscopy, steady-state spectroscopy and computational methods to elucidate the differing functionalisation employed by nature within the MAA family to achieve photostability. Our interest in studying usujirene and palythene stems from the fact these two MAAs (i) are distinct, due to the addition of the conjugated double bond, and may relax via an alternative mechanism compared with other MAAs and (ii) absorb further into the UVA than most MAAs, a spectral region of which there is a lack of UV filters [30]. Furthermore, it is important to add that efficient molecular photon-to-heat conversion does not solely benefit the cosmetic industry; there are pharmaceutical, energy and environmental uses for such molecules [31,32,33].

## 2. Materials and Method

### 2.1. Extraction and Purification

A total of 10 g of commercial dried *P. palmata* flakes (The Cornish Seaweed Company, Cornwall, UK) were left to macerate overnight at 4 °C in a mixture of 25:75 HPLC-grade methanol to HPLC-grade water. The next day, the sample was sonicated for 5 cycles of 45 s with 1 min intervals, followed by centrifugation and recovery of the supernatant which was afterwards dried using a centrifugal evaporator to yield ~2 g of crude extract. The crude extract was resuspended in HPLC-grade water and filtered using a 0.2 µm centrifugal filter for HPLC purification. 

Reverse-phase HPLC was performed using a PrepHT XDB-C18 column (21.2 mm × 100 mm, particle size 5 μm) connected to a 1260 HPLC equipped with a binary pump and diode-array detector (Agilent Technologies, Santa Clara, CA, USA). Injection volumes of 200 μL were used with the following conditions: an isocratic hold of 5% B for 15 min increasing to 10% B for 10 min; then increasing to 100% B over 5 min; and finally, 100% B for 10 min (solvent A = water; solvent B = methanol) with a flow rate of 5 mL/min. The fractions absorbing at 357 nm were collected using an automated fraction collector and dried using a centrifugal evaporator afterwards to yield ~1 mg of usujirene/palythene. Ultra-High-Performance Liquid Chromatography—High Resolution Mass Spectrometry (UHPLC-HRMS) and NMR analysis were used to confirm the collected fractions as the *cis-trans* isomers usujirene and palythene. Details can be found in Sections S1 and S2 of the Supplementary Information (SI), respectively.

### 2.2. Transient Electronic Absorption Spectroscopy

The characterisation of the transient electronic absorption setup at the Warwick Centre for Ultrafast Spectroscopy (Coventry, UK) has been reported before [34], but a brief overview will be provided here, including pertinent details related to the present experiment. A ~50 μM aqueous preparation of the usujirene and palythene mixture, herein referred to as the usujirene/palythene aqueous solution, was circulated through a demountable liquid cell (Harrick’s Scientific, Pleasantville, NJ, USA) to ensure that a fresh sample interacted with each laser pulse pair. Two 950 μm PTFE spacers were sandwiched between CaF_2_ windows (front window 1 mm and back window 2 mm thickness); this spacer size was chosen so that the sample absorbance was below 0.5.

The wavelength of the pump pulses generated by a tunable optical parametric amplifier (TOPAS-prime with UV extension, Light Conversion, Vilnius, Lithuania) was 357 nm, which was the peak absorption for usujirene. The power of the pump pulses was set to 500 μW. The probe, a white light continuum spanning the spectral range 320–720 nm, was generated by focusing a portion of the fundamental 800 nm onto a vertically translating CaF_2_ crystal. Prior to white-light generation, the 800 nm pulse pathlength can be varied to give pump-probe time delays (Δ*t*) between −1 ps and 3 ns using a gold retroreflector mounted onto a motorised optical delay line. Before reaching the sample, the pump beam passes through an optical chopper operating at a repetition rate of 500 Hz, thereby blocking every other pulse of the 1 kHz pulse train. This allows for a direct comparison of the signal detected by a fibre-coupled spectrometer (AvaSpec-ULS1650F, Avantes, Apeldoorn, The Netherlands) for the pump-on and pump-off samples, which is displayed as changes in optical density (ΔOD) in the resultant transient absorption spectra (TAS).

Collated spectra were subsequently chirp corrected for visual purposes using the software package KOALA [35], and fit with global target analysis using the software package Glotaran [36,37]. The K-matrix function was used to fit two sequential kinetic models separately as we believe there are two different dynamical processes present in our TAS. The first $(A\overset{\tau_{\mathrm{FC}}}{\to }B\overset{\tau_{\mathrm{CI}}}{\to }C\overset{\tau_{\mathrm{VC}}}{\to }D\overset{\tau_{\mathrm{PP}}}{\to }E)$ for the photoprotective mechanism and the second $(A\overset{\tau_{\mathrm{FC}}}{\to }F\overset{\tau_{\mathrm{SE}}}{\to }G)$ for the solvated electron dynamics; we discuss these processes in more detail in the discussion (including the SI). We add, however, that we assumed that evolution out of the Franck-Condon region and solvated electron formation occurs with similar lifetimes, so in our fit, τ_FC_ is a combination of both. We acknowledge that there are some consequences to this assumption which we address in more detail in the discussion. Further details on the fitting procedure we have employed using Glotaran can be found in Section S3 of the SI. Additionally, found in Section S3 are the species associated difference spectra (SADS) and residuals, along with the instrument response function of our TAS and power dependency measurements for the excited state absorption and solvated electron absorption features. 

### 2.3. Steady-State Spectroscopy

A ~15 μM usujirene/palythene aqueous solution was irradiated under an arc lamp (Fluorolog 3, Horiba, Kyoto, Japan) at 357 nm with a bandwidth of 5 nm and a power of ~300 μW, which is approximately four times the solar irradiance power at this wavelength and bandwidth. The irradiation period was 120 min and UV-visible spectra were recorded at regular intervals using a UV-visible spectrometer (Cary 60, Agilent Technologies, Santa Clara, CA, USA). This experiment was conducted to closely mimic previous experiments and to help assign any long-term dynamics that persist beyond the final time delay in our TAS (Δ*t* = 3 ns) [24]. A ~20 μM usujirene/palythene aqueous solution was irradiated under a solar simulator (LCS-100 model 94011A, Oriel Instruments, Stratford, CT, USA), the spectrum of which can be found in the SI (Figure S9). The output power of the solar simulator was ~1000 W/m^2^, which is equivalent to one sun at the Earth’s surface. The irradiation period was again 120 min and UV-visible spectra were recorded at regular intervals as above. For both irradiation experiments, the solutions were contained in a 1 cm pathlength quartz cuvette and were shaken regularly in the case of the irradiation under the arc lamp and continuously stirred under the solar simulator due to the setup allowing for the presence of a stirrer plate.

The fluorescence quantum yield of usujirene/palythene was also determined using 9,10-diphenylanthracene as a standard. Full experimental details pertaining to this can be found in the SI (Section S3.6).

### 2.4. Computational Methods

Geometry optimisations using density functional theory (DFT) were performed on usujirene and palythene, both in their neutral and zwitterionic forms, to assess the relative energies of each conformer, as presented Figure 1a. We investigated the isomerisation pathway between usujirene and palythene in their zwitterionic form by rotating the C1-C2-C3-N1 dihedral angle, fixing it at 30° intervals and optimising in the ground state (S_0_) using DFT. The energy of the optimised geometry at each dihedral angle was recorded to gain insight into the energy barrier and potential energy surface between the isomers. These calculations were performed using the software package NWChem at the PBE0/6-311++G** level of theory, with implicitly modelled water using the conductor-like screening model (COSMO) [38,39,40,41,42,43,44,45]. 

Vertical excitation energies of the first five singlet states (S*_n_*) for usujirene and palythene in their neutral and zwitterionic forms were calculated using the second-order approximate coupled-cluster singles and doubles method with the resolution-of-the-identity approximation (RI-CC2) which has previously been shown to closely predict excitation energies for MAAs [15,46,47,48,49]. Predicted UV-visible spectra for zwitterionic usujirene and palythene were plotted using these results by convoluting a Gaussian for each singlet excitation with a fixed width of 0.2 eV. This width was chosen to closely match the experimental UV-visible spectrum. Additionally, for zwitterionic usujirene and palythene the first five triplet states (T*_n_*) were computed using the same level of theory [46,47,48,50,51]. These were performed using the TURBOMOLE software package with the def2-TZVP basis set and in implicitly modelled water using the COSMO solvent model [42,43,44,52,53]. For an additional confirmation of the energy of the T_1_ state, a ΔSCF methodology was used [54]. This was achieved by calculating the single point energy of optimised usujirene and palythene geometries for both the ground singlet (S_0_) and lowest triplet (T_1_) states using DFT at the PBE0/6-311++G** level of theory in implicitly modelled water, and the difference between the two corresponds to the T_1_ energy; NWChem was used for these calculations [38,39,40,41,42,43,44,45]. Most of the computational results from the described calculations can be found in Section S4 in the SI.

A state-averaged complete active space self-consistent field (CASSCF) across two states was performed to examine the relaxation pathways of usujirene and palythene. A (6,5) active space was selected similar to previous studies on alike molecules and more details pertaining to the active space can be found in the SI (Section S4.4) [20,22,55]. The DFT optimised geometries (zwitterionic usujirene and palythene) were reoptimised using state-averaged CASSCF in the ground state (S_0_). These reoptimised geometries were then relaxed in the S_1_ excited state to obtain starting geometries for CI searches which were subsequently performed; note that the S_1_ optimisations did not converge. These calculations were performed in the gas phase with a 6-31G* basis set using the software package Molpro [56,57,58,59,60,61]. 

## 3. Results and Discussion

Previous studies established that the MAA content in red algae, such as *P. palmata*, depends on a range of environmental factors, including the depth at which the algae proliferate and the amount of UV radiation they are exposed to [62,63]. Several samples from different suppliers of edible *P. palmata* flakes were extracted (as described in Section 2) and analysed by HPLC for the presence of usujirene and palythene, see Section S5 in the SI. Variable amounts of usujirene and palythene were detected in the different samples. Different batches from the same supplier also revealed significant variations in MAA content. One specific sample found to be rich in usujirene and palythene was then used to isolate these compounds which were subsequently used in the study.

**Figure 2:**
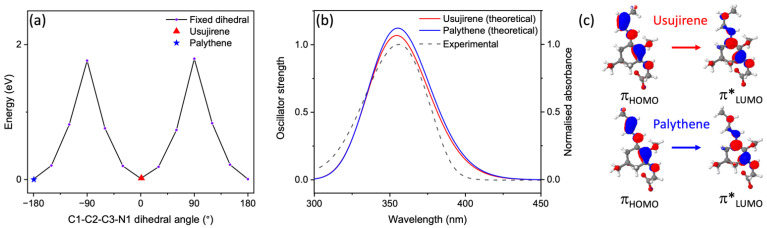
(**a**) Electronic ground state potential energy surface of usujirene to palythene by rotation around the C1-C2-C3-N1 bond computed at the DFT/PBE0/6-311++G** level of theory in implicitly modelled water. (**b**) Predicted UV-visible spectra for usujirene and palythene (solid lines), computed at the RI-CC2/def2-TZVP level of theory in implicitly modelled water, overlaying the experimental UV-visible spectrum (dashed line). (**c**) The orbitals corresponding to the S_1_←S_0_ transition for usujirene and palythene.

The calculated energy and structures of both the neutral and zwitterionic conformers of usujirene and palythene are given in Figure 1, along with the UV-visible spectrum of the usujirene/palythene aqueous solution. From our calculations, it is evident that the zwitterionic forms of both usujirene and palythene are lower in terms of energy compared to their neutral conformer by ~0.80 eV suggesting that the zwitterionic form is the likely structure in aqueous solution. This is supported by previous computational work on other MAAs [18,64]. A comparison between the two isomers found that palythene is the lower energy isomer by only 0.02 eV. We calculated the energy barrier in the electronic ground state between the two isomers to be ~1.80 eV, which is shown in Figure 2a. From this, we conclude that it is unlikely that any interconversion would occur in the solution without being exposed to light or another source of energy.

The peak absorption in Figure 1b is closer to usujirene’s peak absorption (357 nm), which is given by a vertical red line suggesting that it is the more prevalent isomer in our sample. This was confirmed by UHPLC-HRMS, which indicated a ratio of around 3.7:1 usujirene to palythene, see Section S1 in the SI for more details. Whilst this higher prevalence of usujirene could be due to many factors, we tentatively suggest that it may stem from its biosynthetic origin. Usujirene is likely to derive from the decarboxylation of *Z*-palythenic acid, which could in itself result from the syn-elimination of water from porphyra-334. Palythene on the other hand has been proposed to derive from the decarboxylation of *E*-palythenic acid [65].

Our vertical excitation energy predictions of the zwitterionic forms demonstrate that this UVA absorption is due to excitation to the optically bright S_1_ electronic state and the transition has a ππ* character, which is in line with the previous literature on MAAs [15,18]. Figure 2b displays the predicted UV-visible spectra for usujirene and palythene overlaying the experimental spectrum; for an extended spectrum to 200 nm see Figure S11 in the SI. There is considerably good agreement between the predicted spectra and the experimental spectrum. The slight hypsochromic shift for usujirene can be observed in the predicted spectra and this is also observed in the individual experimental spectra obtained for UHPLC-HRMS for usujirene and palythene, see Figure S2. 

From our UHPLC-HRMS and NMR analysis (Sections S1 and S2 in the SI), we can conclude that whilst the sample mostly contains usujirene and palythene, there are some impurities. One identifiable impurity is palythine, a third MAA which results from the hydrolysis of the usujirene/palythene isomeric pair and has a maximum absorption of 320 nm. Furthermore, the peak in the UV-visible spectrum at ~250 nm—see Figure 1b—only partially corresponds to usujirene or palythene and is mostly due to another impurity. This is corroborated by our vertical excitation energy predictions as there are no bright transitions around this region, see Table S3 and Figure S11. In light of this, it is important to highlight that only usujirene and palythene absorb at 357 nm, which is the photoexcitation wavelength used in our TEAS measurements, which we will now discuss.

**Figure 3:**
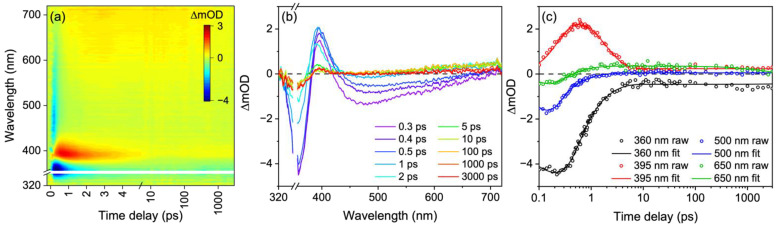
TAS of usujirene/palythene aqueous solution displayed as (**a**) false colour heat map and (**b**) lineouts at selected pump-probe time delays. In (**a**) time delay is plotted linearly until 5 ps and as a logarithmic scale between 5 and 3000 ps. (**c**) Transients at selected wavelengths; the open circles are the raw data and the solid lines are the fit.

TAS following photoexcitation at 357 nm is shown in Figure 3, both as a false colour heat map and as lineouts. Following photoexcitation, there exists a broad stimulated emission across the probe region (410–720 nm) along with a ground state bleach (GSB) feature around 360 nm, both at early Δ*t*. An excited state absorption (ESA) feature around 390 nm grows in shifted from time-zero (Δ*t* = 0, the point at which the pump and probe beams are temporally overlapped). Additionally, there is a faint and broad ESA at the red edge (long wavelengths) of the probe region, which still persists to the maximum Δ*t* (3 ns). Also evident at the maximum Δ*t* is the presence of a GSB and an ESA feature centered at 360 and 390 nm, respectively. Overall, the spectral signatures closely resemble our earlier work on an MAA analogue and the MAAs shinorine and porphyra-334 [15,34].

Our global target analysis fit of the TAS yielded five lifetimes, which are provided in Table 1. Note again that we believe there are two separate sequential kinetic processes occurring in parallel in our TAS. The quality of the fit can be evaluated using the lineouts at selected wavelengths presented in Figure 3c. Additionally, the lifetime assignment and quality of the fit can be assessed using the SADS and residuals, respectively, both of which are found in the SI (Figure S5).

**Figure 4:**
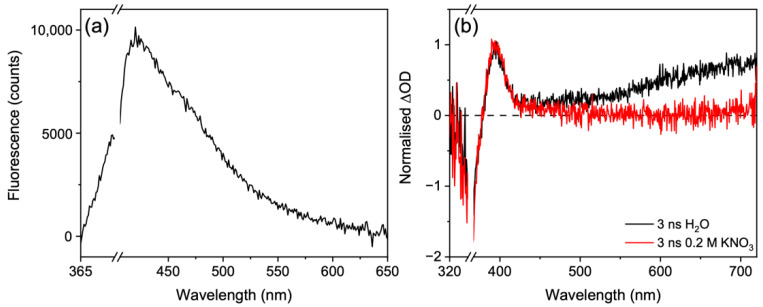
(**a**) Fluorescence spectra of a ~1 μM usujirene/palythene aqueous solution. (**b**) TAS taken at 3 ns of usujirene/palythene in water (black) and in 0.2 M KNO_3_ (red).

With knowledge of the extracted lifetimes and guidance from previous results, we can begin to assign the lifetimes to dynamical components [15,34]. The first lifetime, τ_FC_ = 110 fs, is assigned to the population undergoing a geometry rearrangement (both solute and solvent) as it traverses the excited state potential energy surface out of the Franck-Condon region *en*-route to an energetically accessible S_1_/S_0_ CI. This is evidenced by the decay of the stimulated emission feature across the probe window (410–720 nm) which slightly red-shifts with time. The (albeit very weak) fluorescence spectrum given in Figure 4a closely resembles the stimulated emission, further supporting this assignment. The second lifetime, τ_CI_ = 390 fs, corresponds to the population traversing through the S_1_/S_0_ CI to populate a vibrationally hot electronic ground state. This population will vibrationally cool with a lifetime of τ_VC_ via intermolecular energy transfer from the solute to the solvent. This assignment agrees well with the previously reported work along with our own TAS which shows a sharp blue-shift indicative of vibrational cooling [15,34]. Furthermore, we draw confidence in this assignment given that the ESA and GSB recover on the same timescale, see Figure 3c, and that the ESA is red-shifted and on the edge of the GSB. We attribute the quick vibrational cooling that was observed to the strong solute-solvent interaction due to the large number of hydrogen bonds available with water molecules, which is further strengthened by the charged nature of these zwitterionic molecules within the polar nature of the solvent [15,19]. Such rapid vibrational cooling highlights how efficient nature is at protecting itself from UV radiation. 

Along with the fast dynamics described, there are clear long-lived components evident; firstly, in the GSB and associated ESA at around 360 and 390 nm, respectively, and secondly in the ESA at the red edge of the probe region, at around 700 nm. In our previous work, we proposed that the ESA at the red edge was likely due to solvated electron given its spectral shape [15,66,67]. Here, we demonstrate this more conclusively by quenching the solvated electron with potassium nitrate [68,69], see Figure 4b. Our choice of potassium nitrate over hydrochloric acid was because we wanted to keep the solution under neutral pH as the acidic conditions would likely promote the hydrolysis of usujirene/palythene to palythine. To assess the source of the solvated electron, solvent alone TAS at long Δ*t* were taken. No signal above baseline was observed for the solvent alone, see Figure S7 in the SI, indicating that the solvated electron is likely generated in the solute; therefore, a usujirene or palythene radical cation will also be present in solution. 

We now briefly return to discuss the first lifetime, τ_FC_ = 110 fs, which also incorporates solvated electron formation. We acknowledge that the assumption that solvated electron formation and the evolution out of the Franck-Condon region occurs on the same lifetime is unlikely to be true. The ejection of the electron to form a solvated electron may be instantaneous or may have a lifetime which depends on the excitation wavelength, as was found in a study on phenol [70]. Here, we are unable to distinguish between the two, given that the stimulated emission signal and the solvated electron signal are convoluted over the same region. However, the decay of the stimulated emission signal and rise of the solvated electron signal appears to occur on, or around, the same timescale in our TAS, warranting the decision to encompass both processes within the same lifetime, τ_FC_. We reiterate that τ_FC_ is a combination of the two processes and we cannot determine individual rates for each process. Furthermore, we add that whilst these solvated electron dynamics are certainly interesting, a power-dependency study highlighted that the solvated electron formation is a two-photon process (see Section S3.4 in the SI). Furthermore, multiphoton processes are unlikely to occur in nature and only occur here given that the photon density of our pulsed laser is much greater than a continuous light source (e.g., solar irradiance). Given this, further analysis of the solvated electron formation is outside the scope of this work.

From the GSB feature, we estimate that there is ~85% recovery of usujirene/palythene within 3 ns, with the leftover GSB likely a result of a convolution of processes. Firstly, the geminate pair to the solvated electron tentatively assumed to be a usujirene or palythene radical cation, will contribute towards the persistent GSB. Additionally, the persistent GSB is likely a result of photoproduct formation, possibly from the isomerisation of usujirene to palythene (albeit unlikely given the low reported quantum yield) [24], and trapped population in the electronic excited state either as a singlet or triplet state. The ESA centered at 390 nm could correspond to either photoproduct or trapped electronic excited state population and we return to discuss this below.

Two lifetimes were required to model these long-lived components with one lifetime covering the 320–500 nm spectral region and modelling the photoproduct (τ_PP_) and the other covering the 500–720 nm spectral region and modelling the solvated electron recovery (τ_SE_). The lifetime τ_PP_ > 3 ns, and this is evidenced by the persistent nature of the GSB and ESA which is present from ~5 ps and extends beyond the final time delay with no changes in intensity nor spectral shape. The lifetime τ_SE_ is around 2 ns. From the selected transient with the fit overlaid at 650 nm in Figure 3c (green trace), it is clear that the signal recovers at long Δ*t*. We would expect to see the GSB feature recover along with the solvated electron signal decay if the radical cation recombined with the solvated electron to reform the starting zwitterionic forms. As this is not the case, we tentatively assume that the solvated electron (at least partially) recombined with a different species in our solution. We again reiterate that solvated electron formation in this work is a result of two-photon absorption and is very unlikely to occur in a more real-life environment. As a result, we do not explore these results any further. 

**Figure 5:**
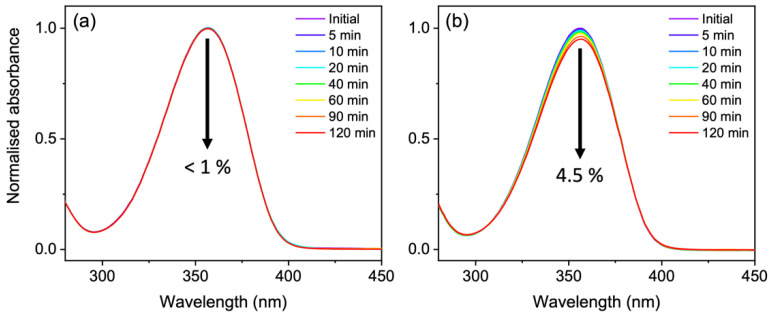
(**a**) UV-visible spectra over 120 min of continuous irradiation at 357 nm of a ~15 μM usujirene/palythene aqueous solution. (**b**) UV-visible spectra over 120 min of continuous irradiation under a solar simulator of a ~20 μM usujirene/palythene aqueous solution.

Previous long-term photostability measurements by Conde et al. found very little photodegradation quantum yields for usujirene and palythene in the order of 10^−5^ after irradiation with monochromatic light at 366 nm [24]. We wanted to mimic a similar experiment using monochromatic light centered at 357 nm and after 120 min of irradiation we observed very little degradation (<1%) over the UVA region in agreement with Conde et al. [24], see Figure 5a. To examine the isomer pair photostability even further, we irradiated a solution under a solar simulator shown in Figure 5b. In this instance, we observed a 4.5% drop over the UVA region after 120 min of irradiation. 

The UV-visible difference spectra (pre- and post-irradiation) for monochromatic irradiation at 357 nm does not resemble the TAS at 3 ns in 0.2 M potassium nitrate (not shown as signal is within error of our UV-visible spectrometer). As a result, we believe the absorption feature at ~390 nm (see Figure 4b) is unlikely to stem from palythene or another photoproduct formation. The reported experimental quantum yield for isomerisation is relatively small which further supports this [24]. The more likely candidate is the trapped population in the electronic excited state either as a singlet or triplet state. We calculated the fluorescence quantum yield for our sample to be <1% (Section S3.6), therefore, if the trapped population is in the S_1_ state, then the majority of the population is finding its way to a CI beyond 3 ns. Furthermore, given the similar spectral signatures and the higher reported triplet quantum yields compared to the fluorescence quantum yields for other MAAs [15,16,25], we tentatively suggest that the incomplete GSB is most likely trapped population in the triplet state. Our calculations suggest that the T_1_ is closest to the S_1_ energy for the Franck-Condon geometries of usujirene and palythene making it the most likely triplet state to be populated (Table S4).

**Figure 6:**
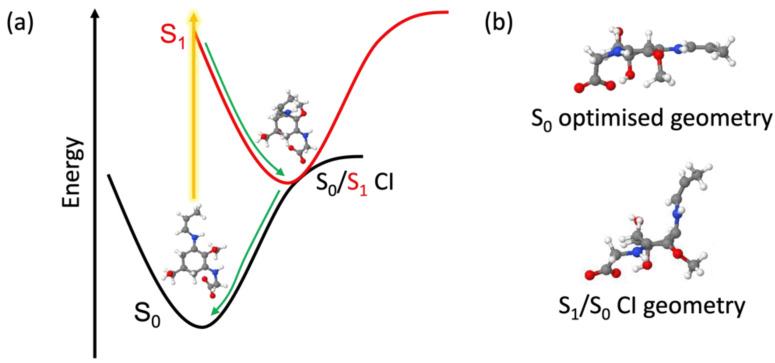
(**a**) Schematic of the dominant relaxation pathway of usujirene with the state-averaged CASSCF/6-31G* optimised S_0_ and S_1_/S_0_ CI geometries. (**b**) The geometries of usujirene in the optimised S_0_ state and at the S_1_/S_0_ CI with the ring along the plane.

Computationally, we investigated the relaxation pathways of usujirene and palythene using state-averaged CASSCF. We allowed unrestricted usujirene and palythene to relax in the S_1_ state and it tended towards a non-planar geometry with the substituent on the ring (containing the additional double bond) folding out of the plane. Starting with this geometry, we were able to locate a CI for each isomer that was very similar in structure, see Figure 6 for usujirene and Table S5 for palythene. We were unable to identify any reasonable geometries along the isomerisation pathway in the S_1_ between usujirene and palythene. As a result, we believe usujirene and palythene relax via the planar to non-planar ring flexing pathway, as has been described for other MAAs and related species [18,20,21,22]—this geometry change can be most evidently observed in Figure 6b. This is further supported by the appearance of the TAS closely resembling our previous work and the reported low quantum yield for isomerisation by Conde et al. [15,24,34].

We close the discussion by adding that whilst the studies presented in this work suggest that usujirene and palythene exhibit impressive photostability and the idealistic properties of a UV filter, we found that these MAAs slowly degraded to palythine over time. Usujirene and palythene are more prone to hydrolysis than other MAAs due to their additional double bond and this may also explain the need to test several sources of *P. palmata* to obtain a sample of usujirene and palythene. As nature is able to synthesise MAAs readily, it is unlikely that this stability is a problem; however, from an industrial application perspective, this may pose a disadvantage, and choosing a more chemically stable and commonly found MAA such as porphyra-334 may be more suitable depending on the desired absorption. Furthermore, investigations into increasing the yield of usujirene and palythene would be a welcomed avenue of research.

## 4. Conclusions

To summarise, the findings from this work reiterate the impressive photostability exhibited by MAAs. Usujirene and palythene, whilst distinct in the MAA family, display similar excited state dynamics to what we found in our previous work for the MAAs shinorine and porphyra-334. Computationally, we found that the photoprotective mechanism likely follows the planar to non-planar ring flexing pathway as with other MAAs due to the fact we were unable to find reasonable geometries along the S_1_ photoisomerisation pathway. Further to this, we extended the steady-state spectroscopic studies of usujirene and palythene to examine their photostability in a more real-life environment. By exposing the MAAs to both UVA and UVB radiation, we found that they remained stable after 120 min. Finally, we highlighted concerns related to the overall stability and availability of usujirene and palythene, which are important considerations for industrial purposes.

## Figures and Tables

**Table 1:** Lifetimes and associated errors extracted from the global fit of the usujirene/palythene aqueous solution TAS.

Lifetime ^1^	τ_FC_ (fs)	τ_CI_ (fs)	τ_VC_ (ps)	τ_PP_ (ns)	τ_SE_ (ns)
	110 ± 70	390 ± 70	1.46 ± 0.07	>3	2.202 ± 0.091

^1^ Franck-Condon (FC), conical intersection (CI), vibrational cooling (VC), photoproduct (PP) and solvated electron (SE).

## Data Availability

The underlying data for this study can be found in the Zenodo data repository at doi:10.5281/zenodo.6242997.

## Supplementary Materials

The following supporting information can be downloaded at: https://www.mdpi.com/article/10.3390/molecules27072272/s1, Figures S1–S3: UHPLC-HRMS analysis; Figure S4: ^1^H NMR spectrum (400 MHz) of usujirene and palythene in D_2_O [23,71]; Figure S5: Species associated difference spectra and fitting residuals; Figure S6: Instrument response function; Figure S7: Solvent-only long time TAS; Figure S8: Power dependency studies; Figure S9: Spectrum output by the solar simulator; Figure S10: UV-visible and fluorescence spectra of usujirene/palythene and 9,10-diphenylanthracene [72,73]; Figure S11: Predicted UV-visible spectra for usujirene and palythene overlaying experimental spectrum; Figure S12: Active space orbitals for usujirene [20,22,55]; Figure S13: Active space orbitals for palythene; Figure S14: UV chromatograms at 360 nm for *P. palmata* extracts; Table S1: K-matrix used for global target analysis fit [35,36,37,74]; Table S2: S_0_ geometries around the C1-C2-C3-N1 dihedral angle; Table S3: Calculated singlet vertical excitation energies for usujirene and palythene; Table S4: Calculated triplet vertical excitation energies for usujirene and palythene; Table S5: Optimised S_0_ and S_1_/S_0_ CI geometries for palythene.
