# Supplementary material for: Investigating the Ultrafast Dynamics and Long-Term Photostability of an Isomer Pair, Usujirene and Palythene, from the Mycosporine-like Amino Acid Family
Source: Molecules. 2022 Mar 31;27(7):2272. doi: 10.3390/molecules27072272 (PMC9000306; doi:10.3390/molecules27072272)
Supplement: Supplementary file 1 [file molecules-27-02272-s001.zip › molecules-1642473-supplementary.pdf]

molecules

MDPI

Article:

Supplementary Information

Investigating the Ultrafast Dynamics and Long-Term Photostability of an Isomer Pair, Usujirene and Palythene, from the Mycosporine-like Amino Acid Family

Abigail L. Whittock [1,2], Jack M. Woolley [1], Nazia Auckloo [1,3], Christophe Corre [1,3] and Vasilios G. Stavros [1,*]

[1] Department of Chemistry, University of Warwick, Coventry, CV4 7AL, UK; abbie.whittock@warwick.ac.uk (A.L.W.); jack.woolley@warwick.ac.uk (J.M.W.); nazia.auckloo@warwick.ac.uk (N.A.); c.corre.warwick.ac.uk (C.C.)
[2] Analytical Science Centre for Doctoral Training, Senate House, University of Warwick, Coventry, CV4 7AL, UK
[3] Warwick Integrative Synthetic Biology Centre and School of Life Sciences, University of Warwick, Coventry, CV4 7AL, UK
* Correspondence: v.stavros@warwick.ac.uk (V.G.S.)

S1. Ultra High-Performance Liquid Chromatography - High Resolution Mass Spectrometry (UHPLC-HRMS) analysis

Part of the dried HPLC fractions containing usujirene and palythene was resuspended in HPLC-grade water and filtered through 0.25 µm filters in preparation for UHPLC-HRMS analysis. The analysis was performed with a 2 µL sample injection through a reverse phase column (Zorbax Eclipse Plus C18, size 2.1 x 100 mm, particle size 1.8 µm) connected to a Dionex 3000RS UHPLC coupled to Bruker Ultra High Resolution (UHR) Q-TOP MS MaXis II mass spectrometer using ESI (Electrospray Ionization in positive mode) with 10 mM sodium formate injected at the start of each run for internal calibration. A *m/z* range of 50-2500 was used. Column elution was done with the following gradient: 0 to 25 % solvent B for 30 mins (solvent A = 0.1 % formic acid in water and solvent B = 0.1 % formic acid in acetonitrile). Using the peak intensities of the UV chromatograms of usujirene and palythene, we were able to predict a ratio of 3.7:1 of usujirene to palythene in our sample.

Palythine: HRMS (ESI-TOF) *m/z*: $[M+H]^+$ Calculated for $C_{10}H_{17}N_2O_5^+$ 245.1132; Found 245.1132; error 0.0 ppm

Usujirene: HRMS (ESI-TOF) *m/z*: $[M+H]^+$ Calculated for $C_{13}H_{21}N_2O_5^+$ 285.1445; Found 285.1452; error 2.5 ppm

Palythene: HRMS (ESI-TOF) *m/z*: $[M+H]^+$ Calculated for $C_{13}H_{21}N_2O_5^+$ 285.1445; Found 285.1448; error 1.1 ppm

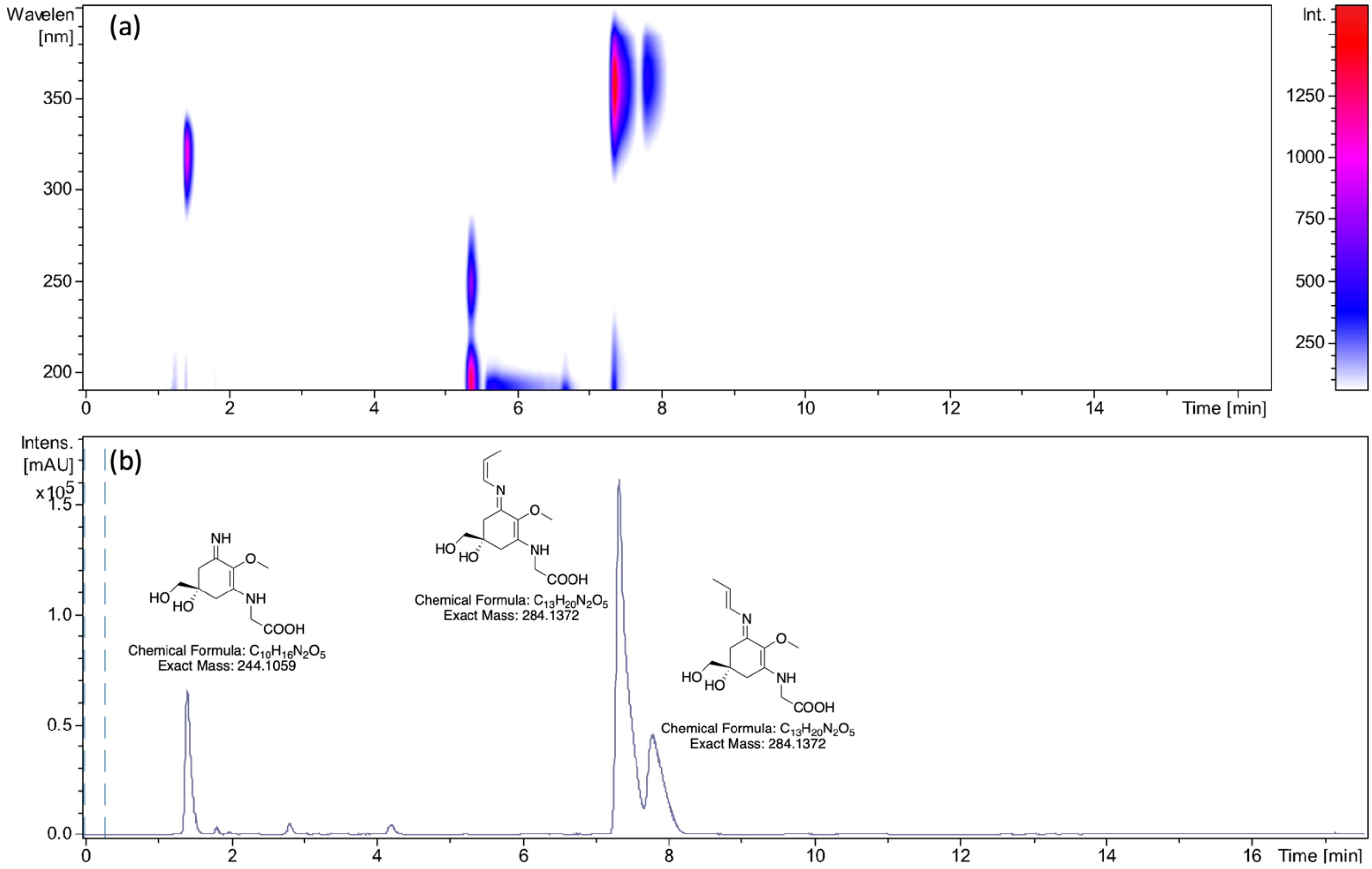

Figure S1. (**a**) 2D spectrum of UV chromatogram vs time. (**b**) UV chromatogram between 300-400 nm with structures of the species corresponding to each peak, namely palythine, usujirene and palythene from left to right.

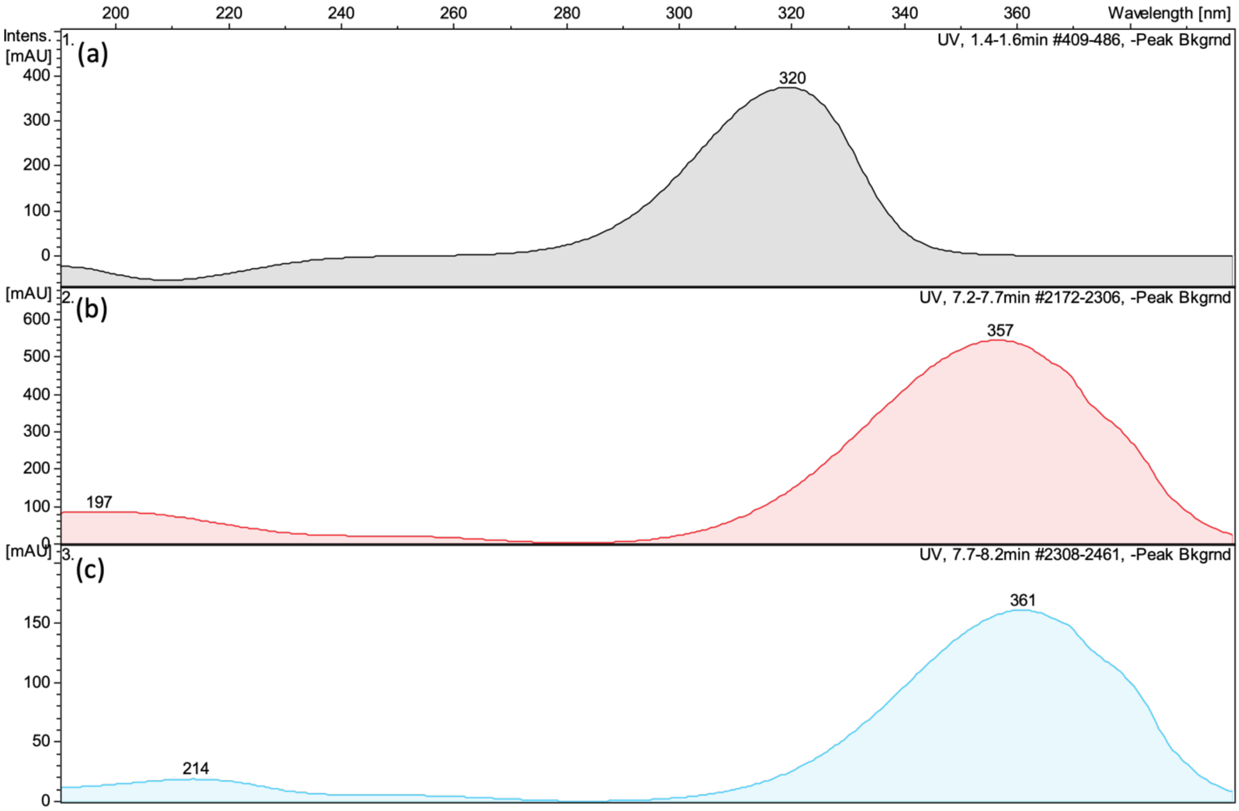

Figure S2. UV chromatograms for (**a**) palythine, (**b**) usujirene and (**c**) palythene.

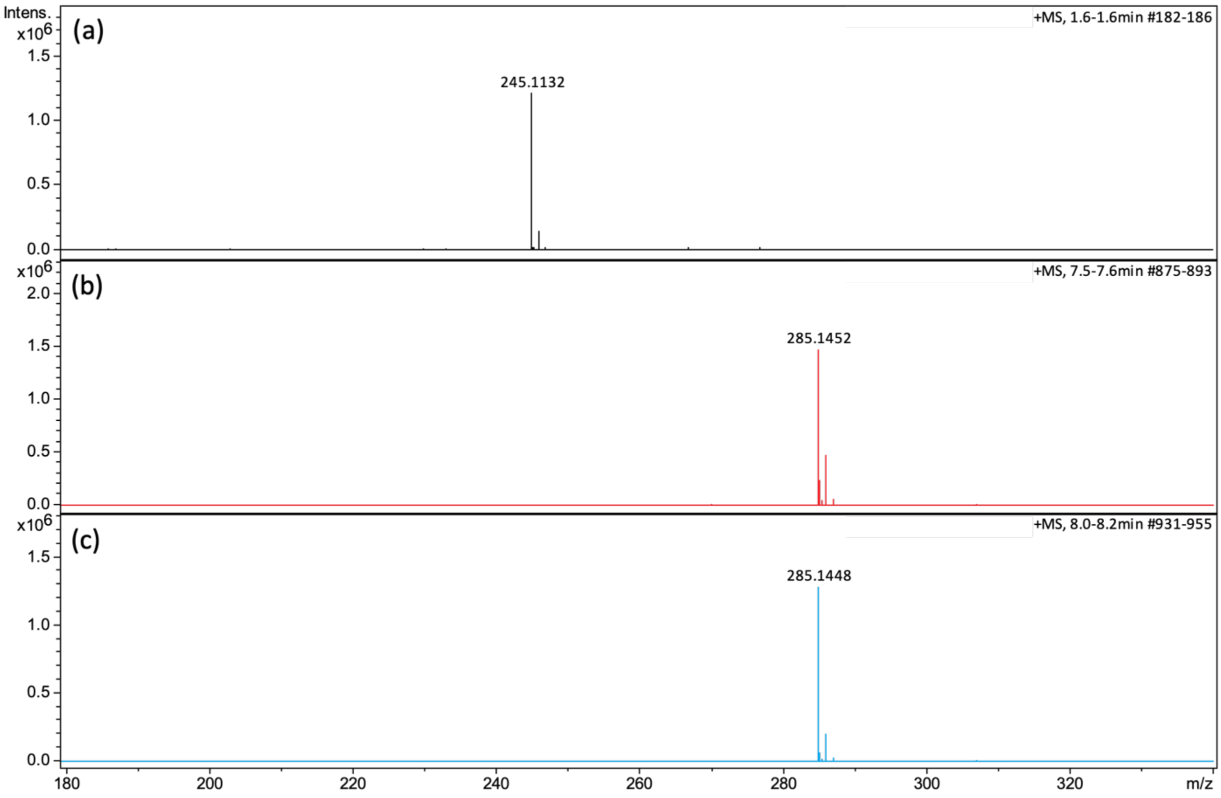

Figure S3. Measured $[M+H]^+$ mass spectra for (**a**) palythine, (**b**) usujirene and (**c**) palythene.

S2. NMR analysis

Part of the dried HPLC fractions containing usujirene and palythene was redissolved in D_2O and a ^1H NMR spectrum was acquired using a 400 MHz NMR spectrometer (Avance III, Bruker). Whilst the sample contains usujirene and palythene, impurities within the sample have been identified evidenced by the UHPLC-HRMS in **Figure S1a** and the ^1H-NMR spectrum in **Figure S4a**. To evaluate the ratio between usujirene and palythene, we focused on the signals corresponding to the methyl groups at position C-13, based on previous assignments [1,2]. A usujurene/palythene ratio of 3.7:1 was calculated by integrating these signals which is consistent with the ratio deduced from the UV chromatograms reported in the main manuscript.

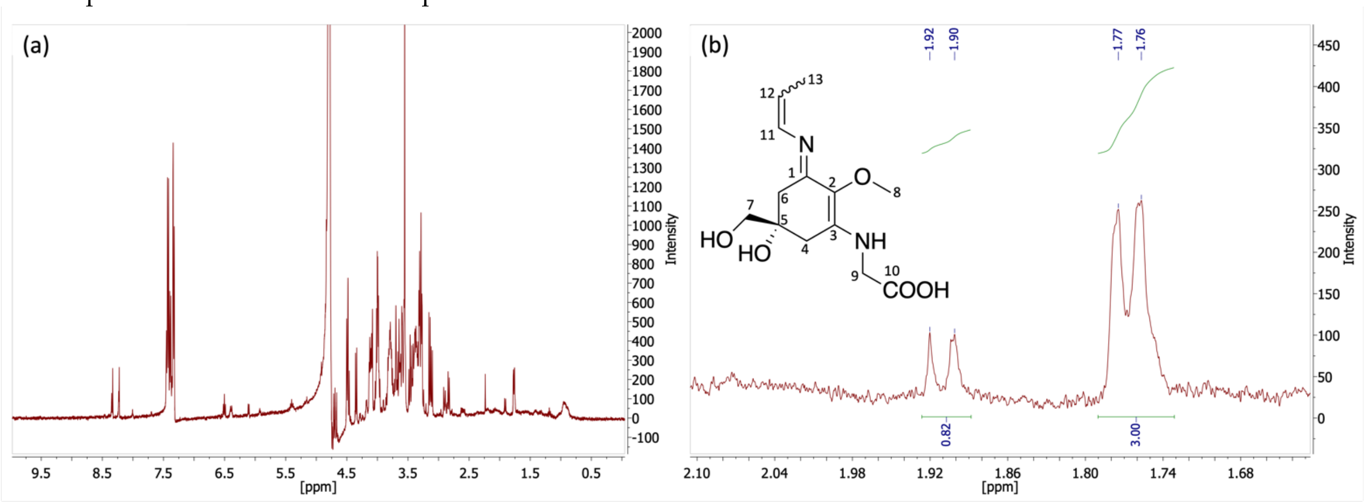

Figure S4. (**a**) ^1H NMR spectrum (400 MHz) of usujirene and palythene in D_2O. (**b**) ^1H NMR spectrum (400 MHz) of usujirene and palythene in D_2O around 1.7-2.1 ppm. Inset is carbon labelled usujirene and palythene structure.

S3. Supplementary experimental information and results

S3.1. Fitting procedure

As discussed in the main manuscript, using the software package Glotaran we fit the transient absorption spectra (TAS) of usujirene and palythene using global target analysis with the use of a K-matrix [3,4], given in **Table S1**, which enabled a sequential kinetic model $(A \xrightarrow{\tau_{FC}} B \xrightarrow{\tau_{CI}} C \xrightarrow{\tau_{VC}} D \xrightarrow{\tau_{PP}} E)$ to fit the photoprotective mechanism and then a second sequential kinetic model $(A \xrightarrow{\tau_{FC}} F \xrightarrow{\tau_{SE}} G)$ to fit the solvated electron dynamics. In short, this model assumes that the lifetime for evolution out of the Franck-Condon region towards the conical intersection and solvated electron formation is similar, and then the two processes, (i) population passing through the conical intersection and vibrational cooling and (ii) solvated electron recovery, occur separately. A discussion on why we believe that modelling τ_{FC} as a combination of the two different processes is given in the main manuscript. We add that species D and E were only modelled between 320 and 500 nm (*i.e.* 500-720 nm was fixed to 0) and species F and G were only modelled between 500 and 720 nm (*i.e.* 320-500 nm was fixed to 0). This was done to compartmentalise and enable two separate lifetimes to be assigned to (i) the photoproduct and persistent ground state bleach which is at the blue edge of the probe and (ii) the recovery of the solvated electron that absorbs at the red edge of the probe. Finally, in order for the fit to converge, τ_{PP} was fixed to a lifetime longer than the final time delay. We justify this because from around 5 ps, the ground state bleach and photoproduct absorption around 360 and 390 nm respectively remain unchanged out to the final time delay of our experiment.

Table S1. The K-matrix used for the global target analysis fit of the usujirene and palythene TAS.

	A	B	C	D	E	F	G
A	0	0	0	0	0	0	0
B	τ_{FC}	0	0	0	0	0	0
C	0	τ_{CI}	0	0	0	0	0
D	0	0	τ_{VC}	0	0	0	0
E	0	0	0	τ_{PP}	0	0	0
F	τ_{FC}	0	0	0	0	0	0
G	0	0	0	0	0	τ_{SE}	0

Due to group velocity dispersion artefacts [5], the TAS are chirped, *i.e.* $\Delta t = 0$ is different for each probe wavelength (λ_{pr}). In order to account for this in our fitting, a third order polynomial is included within the fitting algorithm. Further to this, the fitting algorithm convolutes the Gaussian IRF (see Section S4.3) with exponential functions to extract the fitted lifetimes. We also note that in the false colour heat map and lineouts of the TAS of usujirene and palythene in the main manuscript, the KOALA package has been used to correct the chirp [6].

S3.2. Species associated difference spectra (SADS) and residuals

The averaged percentage error for the residuals from around time zero, $\Delta t = 0$, is approximately 15 % for the residuals given below in **Figure S5b**.

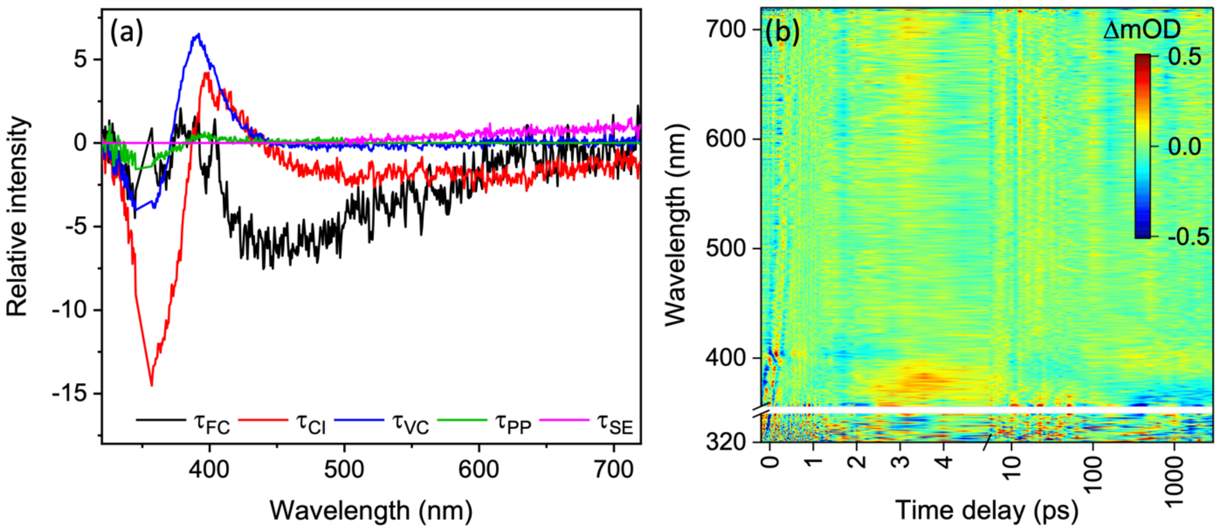

Figure S5. (**a**) SADS for usujirene and palythene photoexcited at 357 nm. (**b**) False colour heat map of the fitting residual for usujirene and palythene photoexcited at 357 nm. Time delay is plotted linearly until 5 ps and then as a logarithmic scale from 5 to 3000 ps. The fitting residual has not been chirp corrected.

S3.3. Instrument response function and solvent only TAS

We conducted solvent-only transients of water following photoexcitation at 357 nm to obtain the instrument response function which determines the limiting temporal resolution of our TAS. The value of the temporal resolution was acquired by fitting a Gaussian over the time-zero response of solvent-only scans and taking the full width half maximum (FWHM). Some of the quoted errors in **Table 1** of the main manuscript are half of the FWHM of the instrument response function rounded to the nearest 10 fs. Additionally, the FWHM of our instrument response function provides an initial guess for one of the input parameters within Glotaran described above (which is subsequently allowed to iterate).

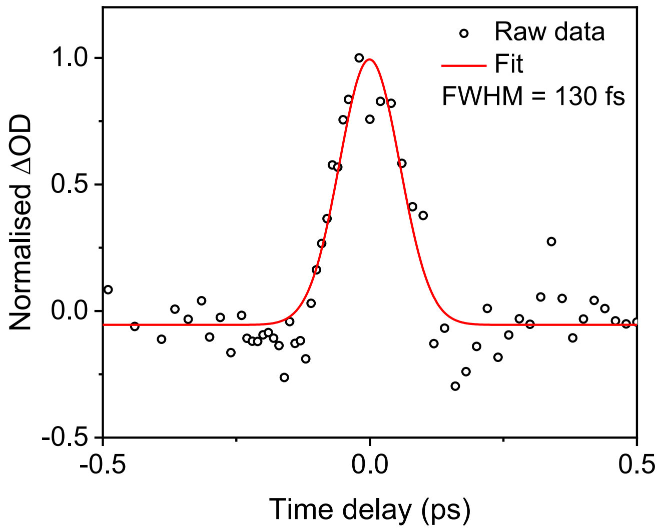

Figure S6. Averaged transient (420-423 nm) for solvent-only time-zero response of water photoexcited at 357 nm (black circles) with a Gaussian fit overlaid (red line); the returned FWHM is ~130 fs and corresponds to the FWHM of the instrument response function.

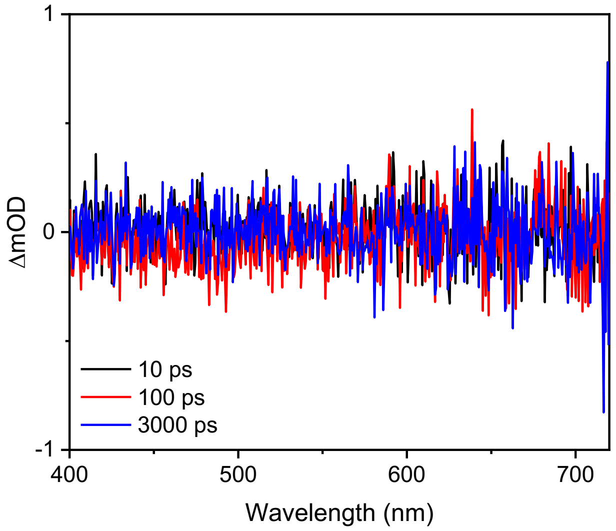

Figure S7. TAS taken at 10, 100 and 3000 ps for water photoexcited at 357 nm from the solvent-only long time scans.

S3.4. Power dependency study

The power dependence of the excited state absorption feature around ~390 nm and the solvated electron absorption feature around ~700 nm was determined. This was achieved by varying the output power of the TOPAS-prime to several powers around the power used for the transient scans. 10 nm integration windows were selected (390-400 nm and 690-700 nm) at a specified Δ*t* (1ps). The gradient of the log(Signal) *vs.* log(Power) plot was around 1 and 2 respectively for each integration window, as can be seen in **Figure S8**, indicating that the solvated electron dynamics we have observed within this work is the result of multiphoton induced dynamics. We acknowledge that this power range is small, however, it has been conducted to obtain a qualitative idea as to the power dependence of the solvated electron.

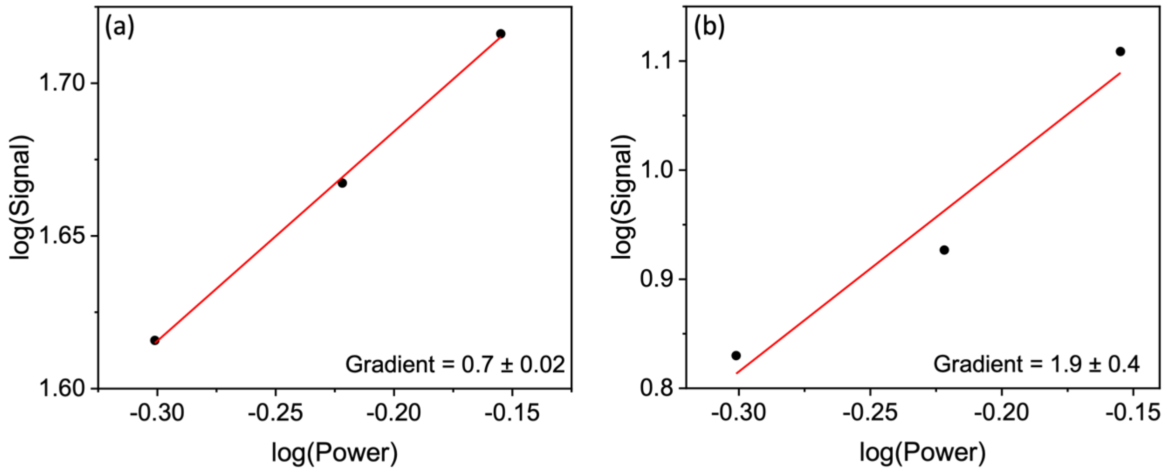

Figure S8. Power dependency of usujirene/palythene aqueous solution photoexcited at 357 nm for (**a**) the excited state absorption at a Δ*t* of 1 ps and an integration window between 390 and 400 nm and (**b**) the solvated electron at a Δ*t* of 1 ps and an integration window between 690 and 700 nm.

S3.5. Solar simulator spectrum

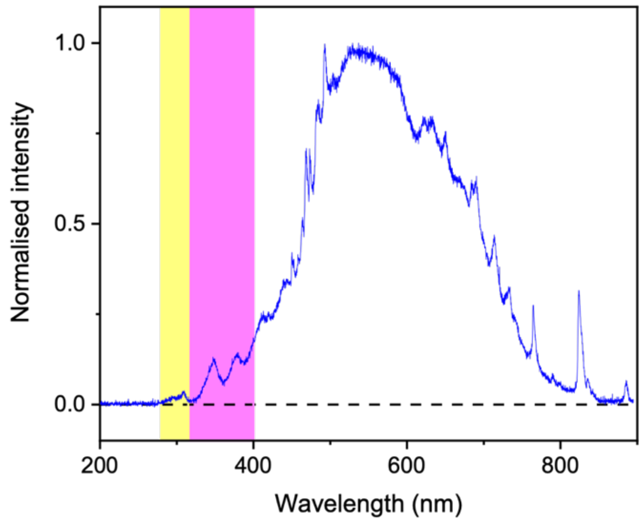

Figure S9. The spectrum output by the solar simulator used for the irradiation experiment in this work. Highlighted in pink is the UVA region (400-315 nm) and highlighted in yellow is the UVB region (315-280 nm).

S3.6. Fluorescence quantum yield

Following the methodology described by Würth *et al.* [7], the fluorescence quantum yield of usujirene and palythene, $\Phi_{f,UP}$, was determined in water by comparing the fluorescence emission to that of a known standard, 9,10-diphenylanthracene (DPA) in cyclohexane ($\Phi_{f,DPA}$ = 0.97) [8]. Both solutions were prepared so that they had an absorbance value of 0.1 or below at the excitation wavelength (357 nm), which corresponds to a concentration of ~1 μM for usujirene and palythene in water and ~9 μM for DPA in cyclohexane. The UV-visible spectra of the sample and standard solutions are shown in **Figure S10a**. Five repeats of the emission spectra were taken for each of the fluorescence samples. These five emission spectra were averaged, shown in **Figure 10b**, and this average was used for the final calculation of $\Phi_{f,UP}$ using Eq. S1;

$$\Phi_{f,UP} = \Phi_{f,DPA} \, {}^{F_{UP}}\!/_{F_{DPA}} \cdot {}^{f_{DPA}}\!/_{f_{UP}} \cdot {}^{n_{UP}^2}\!/_{n_{DPA}^2} \qquad \text{Equation S1}$$

where Φ_f is the fluorescence quantum yield, F is the integral photon flux and n is the refractive index at the wavelength corresponding to half the total integration of the fluorescence emission spectra. $f = 1 - 10^{-A}$, where A is the absorbance at the excitation wavelength. The calculated fluorescence quantum yield of usujirene and palythene ($\Phi_{f,UP}$) was determined to be < 1 %.

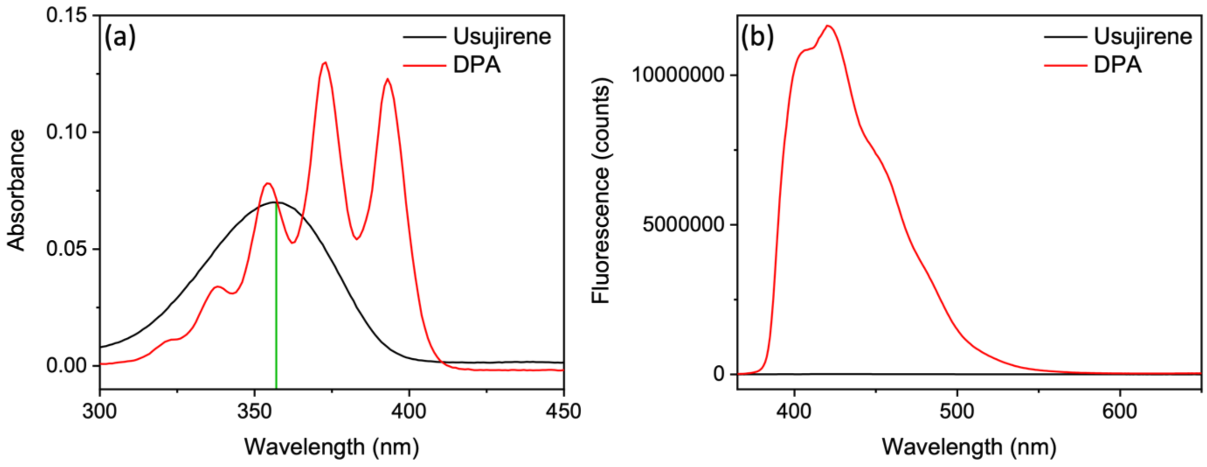

Figure S10. (**a**) UV-visible spectra of ~1 μM usujirene and palythene in water (black line) and ~9 μM 9,10-diphenylanthracene (DPA) in cyclohexane (red line). The vertical green line is at 357 nm which was the excitation wavelength used for acquiring fluorescence spectra. (**b**) Averaged fluorescence spectra of the same samples as in (**a**), attained from averaging 5 separate scans, which were integrated and the values substituted into equation S1.

S4. Supplementary computational results

S4.1. Relaxed geometries around dihedral angle

Table S2. Relaxed S_0 geometries around the C1-C2-C3-N1 dihedral angle along with the relative energy at each angle (relative to unrestricted zwitterionic palythene in the S_0 state, the lowest energy conformer) computed at the DFT/PBE0/6-311++G** level in implicitly modelled water.

C1-C2-C3-N1 dihedral angle (°)			
-180	-150	-120	-90
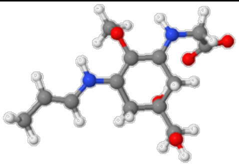<br>0.01 eV	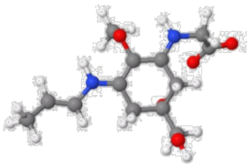<br>0.21 eV	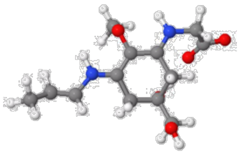<br>0.82 eV	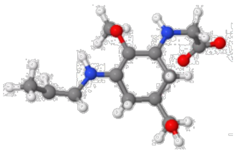<br>1.76 eV
-60	-30	0	30
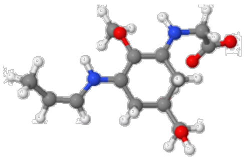<br>0.76 eV	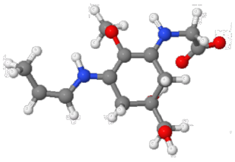<br>0.20 eV	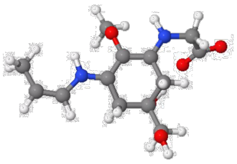<br>0.02 eV	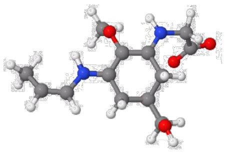<br>0.19 eV
60	90	120	150
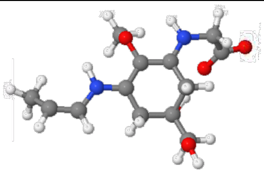<br>0.73 eV	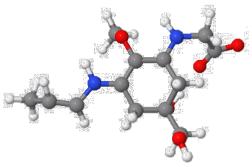<br>1.79 eV	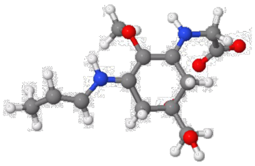<br>0.83 eV	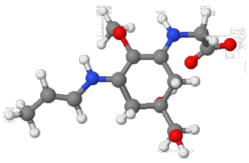<br>0.22 eV
180			
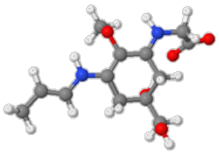<br>0.01 eV			

S4.2. Vertical excitations and relative energies

Table S3. First three singlet vertical excitation energies calculated for the optimised S_0 geometries of usujirene and palythene in their neutral and zwitterionic conformers in implicitly modelled water at the RI-CC2/def2-TZVP level. Also reported are the relative S_0 energies of the conformers (relative to zwitterionic palythene in the S_0 state, the lowest energy conformer) computed at the DFT/PBE0/6-311++G** level in implicitly modelled water.

Conformer	Structure and relative energy	State	Energy (eV)	Energy (nm)	Oscillator strength	Character
Usujirene (neutral)	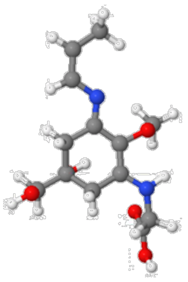 0.80 eV	S_1	3.95	314	1.2089	ππ*
		S_2	4.40	282	0.0085	nπ*
		S_3	5.56	223	0.1024	ππ*
Usujirene (zwitterion)	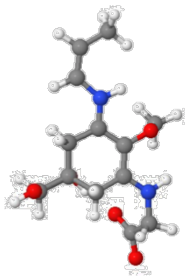 0.02 eV	S_1	3.50	355	1.0685	ππ*
		S_2	4.87	254	0.0060	ππ*
		S_3	5.13	241	0.0228	ππ*
Palythene (neutral)	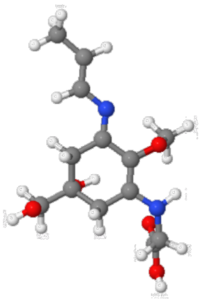 0.77 eV	S_1	4.02	308	1.2438	ππ*
		S_2	4.51	275	0.0091	nπ*
		S_3	5.57	223	0.1124	ππ*
Palythene (zwitterion)	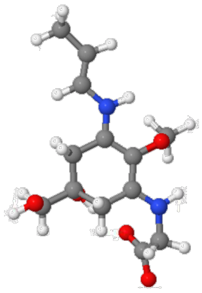 0.00 eV	S_1	3.49	355	1.1232	ππ*
		S_2	4.91	253	0.0072	ππ*
		S_3	5.17	240	0.0193	ππ*

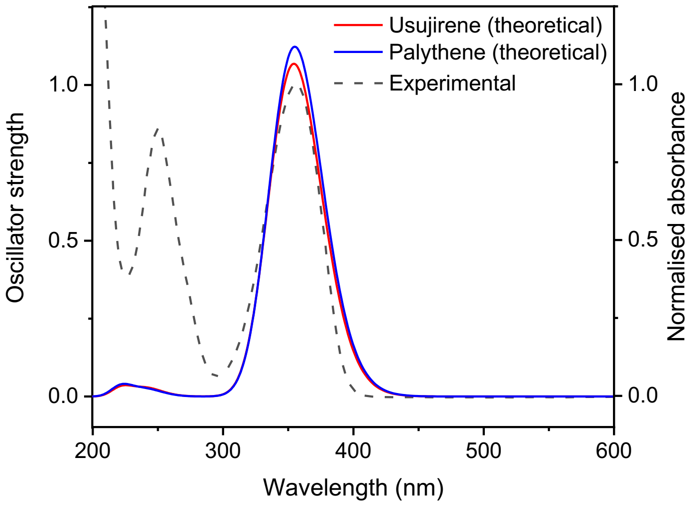

Figure S11. Predicted UV-visible spectra for usujirene and palythene (solid lines) between 200 and 600 nm, computed at the RI-CC2/def2-TZVP level of theory in implicitly modelled water, overlaying the experimental UV-visible spectrum (dashed line).

Table S4. First three triplet vertical excitation energies calculated for the optimised S_0 geometries of usujirene and palythene in their zwitterionic forms in implicitly modelled water at the RI-CC2/def2-TZVP level. ΔT_1 was calculated at the DFT/PBE0/6-311++G** level.

Structure	State	Energy (eV)	Energy (nm)	Orbital character
Usujirene (zwitterion)	ΔT_1	2.40	516	
	T_1	2.76	450	ππ*
	T_2	4.27	291	ππ*
	T_3	4.86	255	ππ*
Palythene (zwitterion)	ΔT_1	2.39	519	
	T_1	2.74	452	ππ*
	T_2	4.24	293	ππ*
	T_3	4.89	254	ππ*

S4.4. Active space orbitals and palythene geometries

A (6,5) active space was selected for the state-averaged complete active space self-consistent field (CASSCF) calculations on usujirene and palythene, in line with previous CASSCF on MAAs and related molecules [9-11]. The chosen orbitals encompassed the π density of the chromophore (π and π* orbitals of the C=C out the ring, C=C in the ring and N=C, and the n orbitals of the N_{imine}, N_{amine} and $O_{methoxy}$). The $S_1 \leftarrow S_0$ ππ* transition is between orbitals 3 and 4 in both usujirene and palythene.

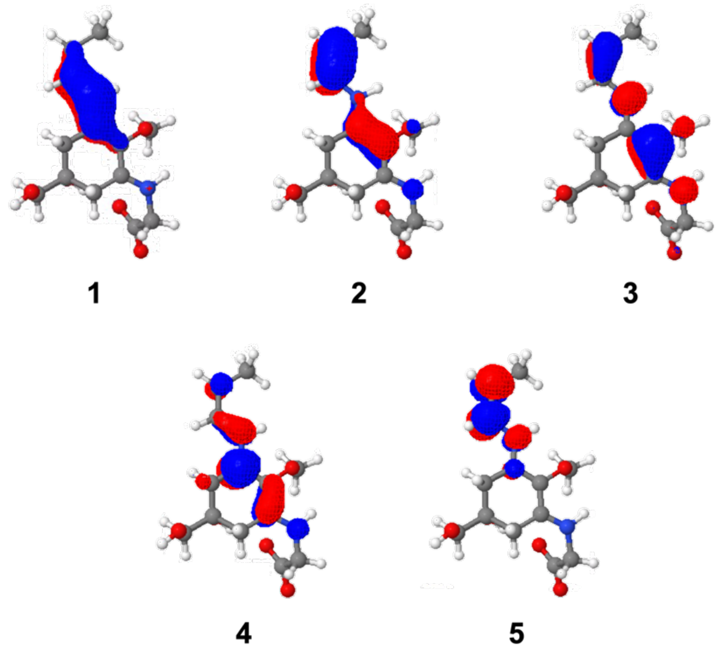

Figure S12. Orbitals involved in the (6,5) active space of usujirene.

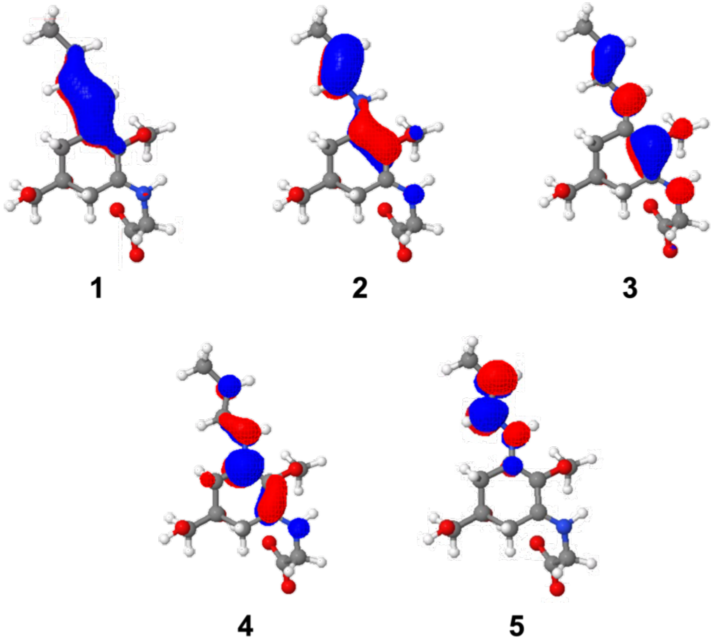

Figure S13. Orbitals involved in the (6,5) active space of palythene.

Table S5. The geometries of palythene in the optimised S_0 state and at the S_1/S_0 CI in two orientations with the ring in and along the plane calculated at the state-averaged CASSCF/6-31G* level of theory.

Plane	S_0 optimised geometry	S_1/S_0 CI geometry
In	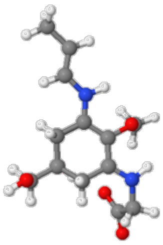	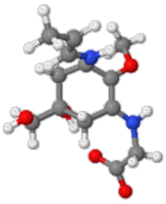
Along	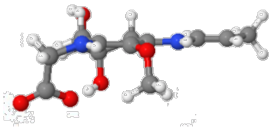	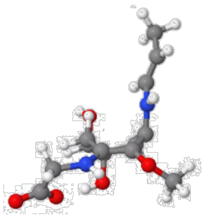

S5. HPLC analysis of *Palmaria palmata* (*P. Palmata*) sources

Given in **Figure S14** are UV chromatograms at 360 nm of *P. Palmata* samples (extracted as described in the materials and methods section in the main manuscript). The batch rich in usujirene/palythene is presented in **Figure S14a** and was the sample subsequently used for purification and the experiments presented in the main manuscript. **Figure S14b** is of an extract from a separate batch from the same supplier and **Figure S14c** is an extract from a different supplier. The conditions for **Figure S14a** are reported in the main manuscript and the elution time for usujirene/palythene is ~14 minutes where there is a clear peak present in the UV chromatogram. The conditions used to acquire **Figure S14b** and **c** was 5 to 25% of solvent B over 30 minutes (Solvent A = 0.1 % formic acid in water and Solvent B = 0.1 % formic acid in methanol) which resulted in an elution time of ~18 minutes for usujirene/palythene. In **Figure S14b**, there is a small peak at this time likely corresponding to a small amount of usujirene/palythene being present and in **Figure S14c** there is no peak in the region indicating that no usujirene/palythene is present. The different conditions used were the result of using an optimised method for purification of the batch containing a high yield of usujirene/palythene (**Figure S14a**) compared to the initial conditions used to verify the presence of usujirene/palythene (**Figure S14b** and **c**).

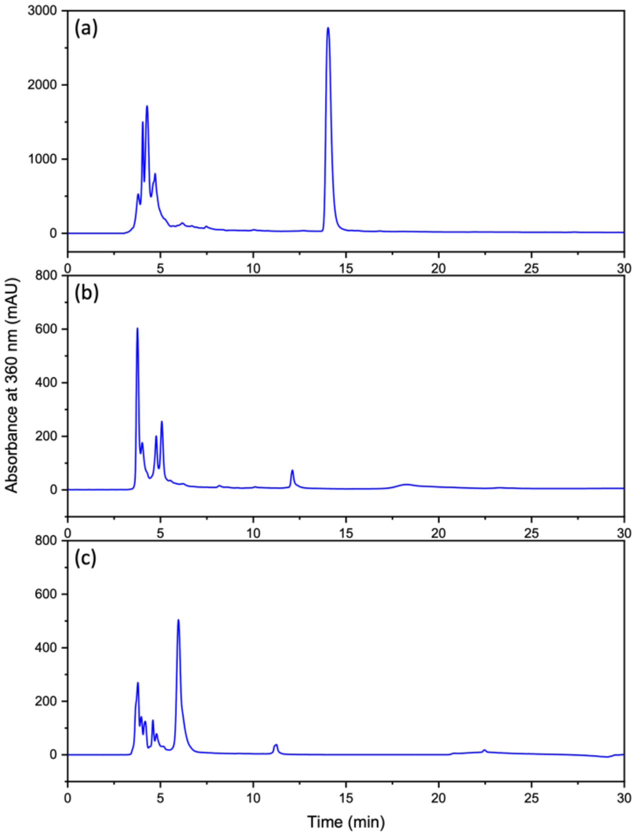

Figure S14. UV chromatograms at 360 nm for *P. palmata* extracts from (**a**) The Cornish Seaweed Company, (**b**) a separate batch from The Cornish Seaweed Company and (**c**) a different supplier.

S6. References

1. Nakayama, R.; Tamura, Y.; Kikuzaki, H.; Nakatani, N. Antioxidant effect of the constituents of susabinori (Porphyra yezoensis). *J. Am. Oil. Chem. Soc.* **1999**, *76*, 649–653.
2. Takano, S.; Uemura, D.; Hirata, Y. Isolation and structure of two new amino acids, palythinol and palythene, from the zoanthid palythoa tubercolosa. *Tetrahedron Lett.* **1978**, *19*, 4909–4912.
3. Mullen, K.M.; Van Stokkum, I.H.M. TIMP: An R Package for Modeling Multi-way Spectroscopic Measurements. *J. Stat. Softw.* **2007**, *18*, 1–46.

4. Snellenburg, J. J.; Laptenok, S.; Seger, R.; Mullen, K. M.; Van Stokkum, I. H. M. Glotaran: A Java-Based Graphical User Interface for the R Package TIMP. *J. Stat. Softw.* **2012**, *49*, 1-22.
5. Walmsley, I.; Waxer, L.; Dorrer, C. The role of dispersion in ultrafast optics. *Rev. Sci. Instrum.* **2001**, *72*, 1-29.
6. Grubb, M. P.; Orr-Ewing, A. J.; Ashfold, M. N. R. KOALA: A program for the processing and decomposition of transient spectra. *Rev. Sci. Instrum.* **2014**, *85*, 064104.
7. Würth, C.; Grabolle, M.; Pauli, J.; Spieles, M.; Resch-Genger, U. Relative and absolute determination of fluorescence quantum yields of transparent samples. *Nat. Protoc.* **2013**, *8*, 1535-1550.
8. Suzuki, K.; Kobayashi, A.; Kaneko, S.; Takehira, K.; Yoshihara, T.; Ishida, H.; Shiina, Y.; Oishi, S.; Tobita, S. Reevaluation of absolute luminescence quantum yields of standard solutions using a spectrometer with an integrating sphere and a back-thinned CCD detector. *Phys. Chem. Chem. Phys.* **2009**, *11*, 9850-9860.
9. Sampedro, D. Computational exploration of natural sunscreens. *Phys. Chem. Chem. Phys.* **2011**, *13*, 5584-5586.
10. Losantos, R.; Funes-Ardoiz, I.; Aguilera, J.; Herrera-Ceballos, E.; García-Iriepa, C.; Campos, P. J.; Sampedro, D. Rational Design and Synthesis of Efficient Sunscreens To Boost the Solar Protection Factor. *Angew. Chem. Int. Ed.* **2017**, *56*, 2632-2635.
11. Losantos, R.; Lamas, I.; Montero, R.; Longarte, A.; Sampedro, D. Photophysical characterization of new and efficient synthetic sunscreens. *Phys. Chem. Chem. Phys.* **2019**, *21*, 11376-11384.
